# *In situ* Parthenogenetic Doubled Haploid Production in Melon “Piel de Sapo” for Breeding Purposes

**DOI:** 10.3389/fpls.2020.00378

**Published:** 2020-04-03

**Authors:** Isidre Hooghvorst, Oscar Torrico, Serge Hooghvorst, Salvador Nogués

**Affiliations:** ^1^Departament de Biologia Evolutiva, Ecologia i Ciencies Ambientals, Secció de Fisiologia Vegetal, Universitat de Barcelona, Barcelona, Spain; ^2^ROCALBA S.A., Girona, Spain

**Keywords:** melon, parthenogenesis, doubled haploid, chromosome doubling, “Piel de Sapo”, X-ray, colchicine

## Abstract

Doubled haploids in cucurbit species are produced through *in situ* parthenogenesis via pollination with irradiated pollen for further use as parental lines for hybrid F1 production. In this study, seven genotypes of melon “Piel de Sapo” were appraised for agronomic traits and pathogen resistances to evaluate its commercial value and used as donor plant material for the parthenogenetic process. Then, *in situ* parthenogenetic capacity of melon “Piel de Sapo” germplasm was evaluated and optimized. Several steps of the parthenogenetic process were assessed in this study such as melon fruit set after pollination with irradiated pollen, haploid embryo obtention, *in vitro* germination and growth of parthenogenetic embryos and plantlets, *in vitro* and *in vivo* chromosome doubling with colchicine or oryzalin and fruit set of doubled haploid lines. Parthenogenetic efficiencies of “Piel de Sapo” genotypes showed a high genotypic dependency during the whole process. Three different methods were assayed for parthenogenetic embryo detection: one-by-one, X-ray and liquid medium. X-ray radiography of seeds was four times faster than one-by-one method and jeopardized eight times less parthenogenetic embryo obtention than liquid medium. One third of melon fruits set after pollination with irradiated pollen contained at least one parthenogenetic embryo. The 50.94% of the embryos rescued did not develop into plantlets because failed to germinate or plantlet died at the first stages of development because of deleterious gene combination in haploid homozygosity. The distribution of the ploidy-level of the 26 parthenogenetic plantlets obtained was: 73.08% haploid, 23.08% spontaneous doubled haploid and 3.84% mixoploid. Two *in vitro* chromosome doubling methods, with colchicine or oryzalin, were compared with a third *in vivo* colchicine method. *In vivo* immersion of apical meristems in a colchicine solution for 2 h showed the highest results of plant survival, 57.33%, and chromosome doubling, 9.30% mixoploids and 20.93% doubled haploids. Fruit set and seed recovery of doubled haploids lines was achieved. In this study, doubled haploid lines were produced from seven donor genotypes of melon “Piel de Sapo,” however, further improvements are need in order to increase the parthenogenetic efficiency.

## Introduction

Melon (*Cucumis melo*) is a eudicot diploid plant species from *Cucurbitaceae*. Melon has been divided in two subspecies, subsp. *melo* and subsp. *agrestis*, and 19 groups have been described by Pitrat (2016): *acidulus*, *agrestis*, *ameri*, *cantalupensis*, *chandalak*, *chate*, *chinensis*, *chito*, *conomon*, *cassaba*, *dudaim*, *flexuosus*, *ibericus*, *inodorus*, *indicus*, *kachri*, *makuwa*, *momordica*, and *tibish*. Together with cucumber (*Cucumis sativa*) and watermelon (*Citrullus lanatus*), melon is one of the most economic important species from *Cucurbitaceae*. Melon production was about 32 million tons in 2017 (FAO, 2017), being China, Turkey, Iran, Egypt, India, Kazakhstan, United States, and Spain, the major producers ordered according to its yield. The melon fruit has a huge genotypic diversity and each country has its own preferences due to cultural reasons (Monforte et al., 2014). *Inodorus* and *Cantalupensis* are the most produced melon groups in Spain. Pathogens are a major threat to melon productivity, Zitter et al. (1996) estimated that over 200 pathogens affected the productivity of cucurbits, caused by fungi, bacteria, viruses or mycoplasma organisms. It is estimated that diseases can cause yield losses of more than 30–50% in melon cultivation (El-Naggar et al., 2012). Powdery mildew, fusarium wilt, and melon necrotic spot virus (MNSV) are the most critical diseases in melon and cucurbit species. Consequently to the high impact of pathogens in cucurbits many modern breeding programs have been implemented to obtain resistant cultivars (Kuzuya et al., 2003; Lotfi et al., 2003).

Commercial seed of melon cultivars can be open pollination (OP) or hybrid F1 cultivar (Robinson, 2000). OP cultivars are inbred lines obtained through several rounds of self-crossing until the obtention of a high homozygous and stable line. On the other hand, hybrid F1 cultivars are stable but heterozygous lines obtained from the cross of two homozygous lines. Hybrids F1 take advantage of heterosis for major fruit yield and pathogen resistances and have a great importance in the European market in spite of its production costs (McCreight et al., 1993; Robinson, 2000). Hybrids F1 are produced by crossing two pure parental lines, which can be obtained by successive rounds of self-crossing and selection during classical breeding or by biotechnology approaches, like doubled haploids (Dong et al., 2016). Doubled haploids (DHs) are pure homozygous lines which require shorter time to produce in comparison to classical breeding (Germanà, 2011). DH lines are generated by androgenesis, gynogenesis or parthenogenesis in major crops, and can be used as a parental for hybrid F1 production or as a stable line. In cucurbit species, *in situ* parthenogenesis through irradiated pollen is the most common and efficient method to obtain haploid plants (Sauton and Dumas de Vaulx, 1987). Those haploid plants need to undergo chromosome doubling using antimitotic compounds. *In situ* parthenogenesis in cucurbits to produce haploid embryos is usually low, from 0 to 5% of seeds contain haploid embryos (Dong et al., 2016), and is less efficient and more time-consuming than other crop species such as: wheat (Niu et al., 2014), bell pepper (Irikova et al., 2011), rice (Hooghvorst et al., 2018), or onion (Fayos et al., 2015).

First haploids of melon, embryos and plants, were obtained by an interspecific crossing with *Cucumis ficifolius* (Dumas de Vaulx, 1979). Then, *in situ* production of haploid embryos was achieved thought pollination with irradiated pollen (Sauton and Dumas de Vaulx, 1987). The pollination of a female flower stigma with irradiated pollen stimulates an *in situ* parthenogenetic response when pollen tube reaches the egg-cell. Then, parthenogenetic haploid embryo is developed, extracted and cultured *in vitro*. Germinated embryo regenerates into a full-developed plantlet that need to undergo chromosome duplication for DH seed recovery. Nevertheless, *in situ* parthenogenesis in cucurbits and specifically, in melon, has many bottlenecks that reduces its efficiency in each step of the process. Melon parthenogenesis has a high genotypic dependency and methodological issues that impede the efficient production of DHs such as: low levels of female flowers developed once pollinated with irradiated pollen; low production of haploid embryos; difficulty to detect seeds containing haploid embryos; low germination of haploid embryos *in vitro*; high mortality of germinated embryos and growing plantlets; very low or null spontaneous chromosome duplication; difficulty to induce chromosome doubling in haploid plants due to a high mortality and hyperhidricity; high ratio of haploid and mixoploid plants; low pollen germination levels of chromosome doubled plants which trigger a decrease of fruit set and seed recovery; and, low DH seed germination (Lim and Earle, 2008, 2009; Gonzalo et al., 2011; Dong et al., 2016).

The seeds of melon fruits set produced via pollination with irradiated pollen are inspected in search of parthenogenetic embryos. The inspection of seeds one-by-one under a stereo microscope is successful and the most commonly applied although its time-consuming and labor-intensive. Two other methods have been reported for parthenogenetic haploid embryo detection such as X-rays, which had been proven efficient but demand high equipment specialization, and liquid culture, which had been proven ineffective (Dong et al., 2016). The low rate of spontaneous chromosome doubling during melon parthenogenesis process require the implementation of a chromosome doubling step using antimitotic compounds. Colchicine has been the most used antimitotic in melon for chromosome doubling, either via immersion of *in vitro* shoot tips or nodular explants, or via immersion of *in vivo* shoot tips. Chromosome doubling rate can range from 0 to 90% depending on the genotype (Yetisir and Sari, 2003; Lim and Earle, 2008; Gonzalo et al., 2011; Solmaz et al., 2011).

The main objective of this study was to evaluate the commercial value and the parthenogenetic capacity of seven genotypes of *C. melo* var. *Inodorus* “Piel de Sapo” type to obtain DH lines which might be further used as parental lines for commercial hybrid F1 seed production. Moreover, the parthenogenetic generation of DHs from the seven genotypes was evaluated and optimized through the analysis and description of the different steps of the process, assaying: three haploid embryo rescue protocols, previously described in the literature; three chromosome doubling methods; and, a new cytometry flow method for evaluating the ploidy-level.

## Materials and Methods

### Plant Material and Growth Conditions

**FIGURE 1:**
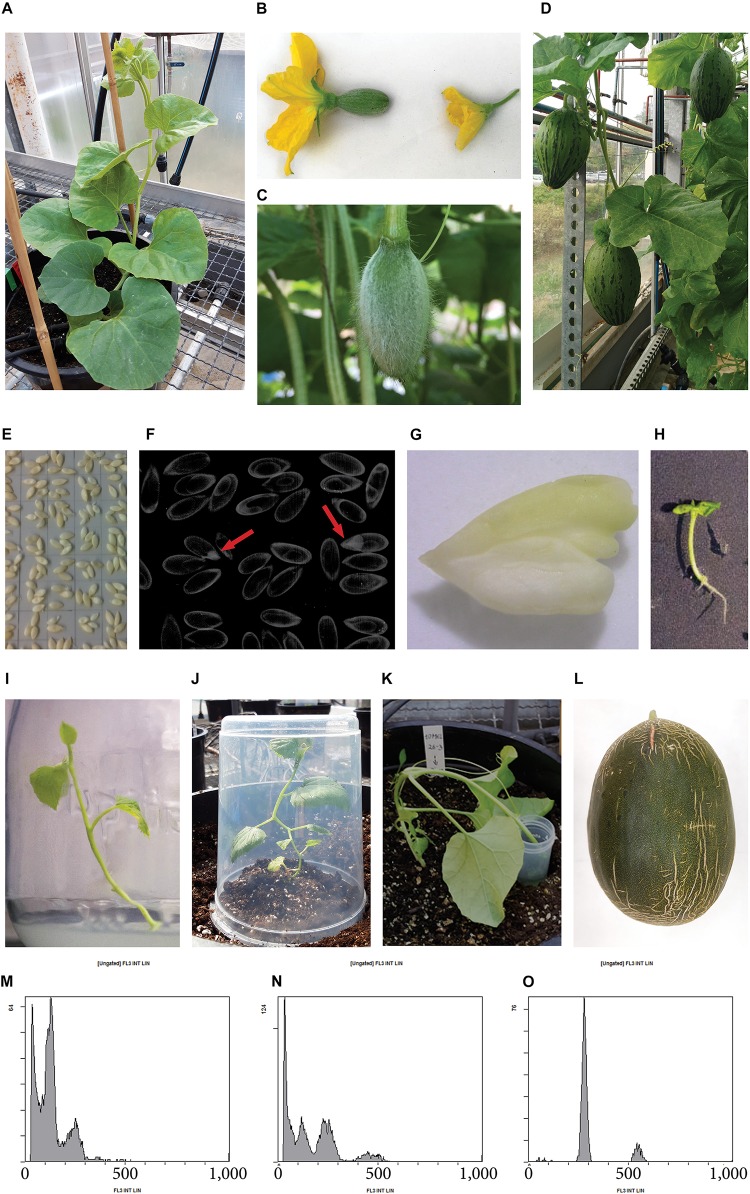
Production of parthenogenetic DH lines in melon “Piel de Sapo” genotype. **(A)** Melon donor plant. **(B)** Detail of a female flower **(left)** and a male flower **(right)**. **(C)** Female flower developed after pollination with irradiated pollen. **(D)** Melon fruits set after 3 weeks of pollination with irradiated pollen, which potentially contain parthenogenetic embryos in their seeds. **(E)** Melon seeds in an acetate sheet ready to be photographed by X-ray. **(F)** X-ray photography of seeds, parthenogenetic embryos are pointed with a red arrow. **(G)** Parthenogenetic embryo rescued. **(H)** Germinated embryo *in vitro*. **(I)** Micropropagated melon plantlet. **(J)** Acclimated melon plant covered with a plastic pot to avoid dryness. **(K)** *In vivo* chromosome doubling treatment of a haploid plant by immersion of the apical meristem into a colchicine solution. **(L)** Melon fruit of a DH line. **(M)** Flow cytometry histogram of a haploid plant, showing ploidy-peaks at channels 150 and 300, corresponding to haploid cells in G0 phase and G2 phase, respectively. **(N)** Flow cytometry histogram of a mixoploid plant, showing ploidy-peaks at channels 150, 300, and 600, corresponding to haploid cells in G0 phase, diploid cells in G0 phase and haploid cells in G2 phase, and, diploid cells in G2 phase, respectively. **(O)** Flow cytometry histogram of a DH plant, showing ploidy-peaks at channels 300 and 600, corresponding to diploid cells in G0 phase and G2 phase, respectively.

Seven genotypes of *C. melo* subsp. *melo* “Piel de Sapo” *indodorus* type were used as plant material (provided by ROCALBA S.A.). Six genotypes were inbred lines (PS-1305, PS-1901, PS-0301, PS-0709, PS-2001, and PS-2301) and one genotype was an open pollinated cultivar (Melito). Melon plants were grown in greenhouse conditions at *Servei de Camps Experimentals* at the *Universitat de Barcelona* (Barcelona, Spain) in 9 L plastic containers filled with substrate containing Floratorf peat moss (Floragard Vertriebs, Oldenburg)—vermiculite (2:1 v/v) substrate supplemented with Osmocote (The Scotts Company LLC, United States) and 1 g CaCO_3_ per peat liter was added to adjust the substrate pH to 6 (Figure 1A).

### Pollination With Irradiated Pollen, Parthenogenetic Embryo Rescue, and Germination

Male flowers containing mature pollen were collected early in the morning and irradiated at 250 Gy using a ^137^Cs source at *Centres Científics i Tecnològics* at the *Universitat de Barcelona* (Barcelona, Spain). Female flowers were emasculated, pollinated with the help of a brush, and bagged to avoid external pollinations. Each female flower receptor was pollinated with three to five irradiated male flowers (Figure 1B). Pollination was done at the same and the next day of irradiation. After 3 weeks, melon fruits that set were harvested and opened for seed collection. Three different methods of seed inspection and embryo detection and rescue were assayed: seed inspection one-by-one with the help of a light box, X-ray radiography of seeds and floating seeds in liquid medium. In the one-by-one individual inspection of seeds method, seeds were sterilized in 20% sodium hypochlorite supplemented with 4 drops⋅L^–1^ of Tween 20, rinsed three times in sterile water and opened one-by-one with the help of a stereo binocular microscope and a light box. In the detection of embryos by X-ray radiography method, seeds were placed in an acetate sheet (Figure 1E) on the Imaging Screen K, introduced in a Faxitron^®^ cabinet X-Ray system (Hewlett Packard, Palo Alto, CA, United States) to be exposed to 16 kV during 70 s and the seeds containing embryo were selected and sterilized (Figure 1F). For liquid culture of seeds, seeds were sterilized and cultured *in vitro* in E20A liquid medium in jars. Detected seeds containing embryos with the three methods were manually opened in aseptically conditions and embryos were cultured *in vitro* in E20A solid medium (Sauton and Dumas de Vaulx, 1987) in 90 mm petri dishes.

### Chromosome Doubling

Three different protocols were assayed to induce chromosome doubling in haploid plantlets, two *in vitro* and one *in vivo*. The two *in vitro* treatments used nodes and shoot tips as plant material, the protocol of Lim and Earle (2009) applied 500 mg L^–1^ of colchicine for 12 h; and, the protocol of Ebrahimzadeh et al. (2018) applied 50 mg L^–1^ of oryzalin for 18 h. Each chromosome doubling method was performed three to five times in different days to treat them as independent replicates. For both *in vitro* treatments, *in vitro* haploid plantlets were micropropagated aseptically, nodes with two to three axillary buds and shoot tips with one to two axillary buds were treated in E20A liquid medium supplemented with the antimitotic solution and DMSO 2% (v/v) during the correspondent exposition time. Once the expositition time elapsed, nodes and shoot tips were rinsed with water and cultured in E20A solid medium. The third chromosome doubling protocol assayed was *in vivo*, following the methodology of Solmaz et al. (2011) and Yetisir and Sari (2003). *In vitro* haploid plantlets were acclimated in the greenhouse (Figure 1J) and when plants expanded four to eight leaves, apical stem was submerged in a 5,000 mg L^–1^ colchicine solution supplemented with 2 drops⋅L^–1^ of Tween 20 for 2 h (Figure 1K). Apical stems were rinsed with water after the treatment.

When apical stems expanded new leaves since the application of the antimitotic treatment, ploidy-level was determined to identify the induced chromosome doubled plants. *In vitro* chromosome doubled plantlets that survived and grew roots were acclimatized in a growth chamber at 25°C, illuminated with 50–70 μmol m^–2^ s^–1^ fluorescent light under a 16/8 h day/night photoperiod and covered with plastic pots. After 2 weeks, the plantlets that survived were acclimatized in the greenhouse (Figure 1J). The stems of *in vivo* treated plants that remained haploid were pruned, and the ones that chromosome doubled were grown until flowering and autopollinated. If plants showed phenotypical mixoploidy, carrying male flowers with and without pollen, stems with haploid flowers were pruned.

### Ploidy-Level Determination

The ploidy-level of germinated parthenogenetic embryos and antimitotic treated plants was determined by flow cytometry following the procedure of Hooghvorst et al. (2018) with slight modifications. About 5 mg of young leaves were collected and put into ice-cold 2 mL microcentrifuge tubes each with a steel bead (3 mm diameter). To each tube, 300 μL of cold lysis buffer (0.1 M citric acid and 0.5% Triton X-100 in distilled water) were added. Tubes were cooled at −20°C for 10 min. Samples were shaken at 25 Hz for a total of 20 s in a MM 400 tissue lyser (Retsch, Haan, Germany) two times and tubes were vortexed with a vortex mixer between the two triturations. The aliquot from each tube was filtered through a 22 μm nylon filter (Sefar Maissa, Blacktown, Australia), gently vacuumed and transferred to a flow cytometry sample tube (Beckman Coulter Inc., Pasadena, CA, United States). Afterward, 150 μL of propidium iodide (PI) stain solution [0.25 mM Na_2_HPO_4_, 10 mL 10× stock (100 mM sodium citrate, 250 mM sodium sulphate) and 9 M PI made up to 100 mL with Milli-Q water] was added to each tube. Tubes were then sealed and kept on ice in the dark for 1 h before flow cytometry analysis. The stained nuclei samples were analyzed using a GalliosTM Flow Cytometer (Beckman Coulter Inc., Pasadena, CA, United States) with a 488-nm laser at the Cytometry Unit (Scientific and Technological Centers, University of Barcelona) and a 32-well carrousel. One diploid control sample was included every seven measurements. Flow cytometry data was analyzed using Summit Software v4.3 (Beckman Coulter Inc., Pasadena, CA, United States).

### DH Haploid Seed Recovery

The acclimated and chromosome doubled plants were grown in the greenhouse. When melon plants flowered, autopollination of DH plants was done by pollinating the emasculated female flowers with three to five male flowers. Once female flowers were pollinated, they were bagged and 3 days later the viability was checked. After 5 weeks since pollination melon fruits set were collected and DH seed was recovered.

### Evaluation of Melon Fruit Traits

Melon fruit traits of donor genotypes were evaluated at commercial maturity stage. The evaluated traits were: fruit weight (FW); fruit diameter (FD); fruit length (FL); fruit shape (FL/FD); fruit skin netting (FSN), evaluated as 0 (no netting), 1 (low netting), 2 (moderate netting), 3 (high netting) and 4 (very high netting); sugar content (°Brix); and, fruit aroma, evaluated as 0 (no aroma), 1 (good aroma), 2 (very good aroma), and 3 (excellent aroma). The fruit aroma evaluation was done by a board of experts. Five to eight melon fruits were evaluated in each genotype.

### Powdery Mildew, Fusarium Wilt, and MNSV Evaluation

The resistance or susceptibility of the seven donor genotypes was evaluated for *Podosphaera xhantii*, *Fusarium oxysporum* f.sp. *melonis*, and, MNSV. Five races of powdery mildew fungi *Podosphaera xhantii* (*Px*) were evaluated 1, 2, 3, 3.5, and 5. Fungi material was provided by GEVES (France). Plants were grown in the greenhouse until expansion of the third true leaf. Leaf disks of 9 cm diameters were taken from the first true leaf, disinfected in 20% sodium hypochlorite supplemented with 4 drops⋅L^–1^ of Tween 20 for 20 s, rinsed twice in sterile distilled water and plated into 90 mm petri dishes filled with powdery mildew medium, 25 mg L^–1^ of Benzimidazole and 1.6 g L^–1^ agar. Each leaf disk was inoculated manually with five conidiophores and up to ten leaf disks were analyzed in each genotype. After 12–15 days upon infection, each leaf disk was phenotypically evaluated according to the sporulation level using a scale from 0 to 4: 0, no sporulation; 1, hyphae present without no conidiophores and yellowing leaf disk; 2, hyphae present and up to twenty conidiophores; 3, 20–100 conidiophores present; 4, more than 100 conidiophores. Level 0 and 1 were considered as resistant, and levels 2–4 were considered as susceptible.

Resistance or susceptibility to *Fusarium oxysporum* f.sp. *melonis* (*Fom*) fungi races 0, 1, and 2 was evaluated. The fungi material was provided by BCCM (Belgium). Plants were grown in the greenhouse until third true leaf was expanded. Then, plants were removed from the substrate, the roots were washed with tap water and soaked for 30 s in a fungi solution of 3 × 10^6^ spores⋅m L^–1^. After the infection, plants were planted again in the substrate. Five plants were analyzed in each genotype. After 30 days upon infection, plants were evaluated according to fusarium wilt symptoms using a scale from 0 to 4: 0, plant without disease; 1, low levels of wilting and yellowing leaves; 2, leaves heavily affected by wilting; 3, all leaves wilted; 4, dead plant. Levels 0 and 1 were considered as resistant, and levels 2–4 were considered as susceptible.

For MNSV evaluation, plants were grown in the greenhouse until the expansion of the third true leaf. Then, virus inoculation was carried out by mechanical inoculation on the cotyledon with a solution of 0.03 M Na_2_HPO_4_, 0.2% DIECA, 75 g L^–1^ carborundum and 1 g of leaf infected with MNSV. Once the inoculation was made, plants were grown at 22°C and 50–70 μmol m^–2^ s^–1^ fluorescent light under a 16/8 h day/night photoperiod. Five plants were analyzed in each genotype. After 20 days upon infection, plants were evaluated according to the presence of virus symptoms using a scale from 0 to 3: 0, no symptoms present; 1, presence of few necrotic spots in the cotyledons; 2, presence of necrotic spots in cotyledons and true leaves, and malformation of new expanded leaves; 4, collapsed plant.

### Statistical Analysis

The parameters FW, FD and FL were analyzed using ANOVA one-way test with a *post hoc* Bonferroni test (*P* < 0.05). The Brix (°) parameter was analyzed with Kruskal–Wallis with a *post hoc* Dunn’s test (*P* < 0.05). The parameters FSN and fruit aroma and the parthenogenesis efficiencies were analyzed with Chi Square test (*P* < 0.05). All experiments were established in a completely randomized design.

## Results

### Evaluation of Parental Donor Genotypes

Agronomic traits of the seven donor genotypes were evaluated in a random designed experiment during two consecutive years, 2017 and 2018 in test fields property of ROCALBA S.A. located in Monzón, Spain (Table 1). The fruit length (FL) of the evaluated genotypes ranged around 23 cm excepting Melito genotype which had a statistically different length (*P* < 0.05) of 19.82 ± 1.35 cm. FD showed significant differences between genotypes (*P* < 0.05), Melito had the smallest diameter and PS-0709 the widest. The shape parameter correlates FL and FD and therefore, express if fruits are ovate, elliptic, or elongated when higher or lower the ratio. The PS-0301 genotype showed the most elliptical melon fruits. The FW of melon fruits was relatively stable inside the same genotype. Nevertheless, significant differences (*P* < 0.05) between genotypes were found, Melito showed the lightest melon fruits (1.83 ± 0.35 kg) and PS-0809 the heaviest melon fruits (3.81 ± 0.77 kg). Sugar content, measured in Brix (°), and aroma are independent, higher values of sugar do not entail a better flavor. Melito and PS-1305 were considered as the best genotypes in terms of flavor because of the high values of aroma and sugar. No significant differences (*P* > 0.05) were found for the Kruskal–Wallis test analyzing FST and aroma due to a low number of replicates and the reduced range of the parameter. Pathogen resistance evaluation showed a high number of resistances in most of the genotypes excepting Melito, which was only resistant to *Fom* race 2 and sensitive to the other pathogens and races assayed. The genotypes PS-1305, PS-0301, PS-0709, PS-2001, and PS-2301 showed resistances for all *Px* and *Fom* races evaluated. However, resistance to MNSV was only present in PS-1305, PS-0709, and PS-2301.

**TABLE 1:** Fruit trait evaluation and pathogen resistance analysis of the seven genotypes of melon “Piel de Sapo.”

	PS-1305	PS-1901	PS-0301	PS-0709	PS-2001	Melito	PS-2301
FL	23.53 ± 20^a^	23.26 ± 1.86^a^	24.88 ± 1.70^a^	23.16 ± 1.94^a^	22.81 ± 3.46^a^	19.82 ± 1.35^b^	23.82 ± 1.72^a^
FD	14.28 ± 1.42^abc^	15.63 ± 1.86^abc^	15 ± 1.65^abc^	17.37 ± 1.82^d^	15.47 ± 2.13^abc^	13.11 ± 1.35^ac^	14.45 ± 1.71^abc^
Shape	1.65	1.49	1.66	1.33	1.47	1.51	1.65
FW	2.58 ± 0.36^a^	3.26 ± 0.69^ab^	2.86 ± 0.50^a^	3.81 ± 0.77^bc^	3.29 ± 1.13^ac^	1.83 ± 0.35^d^	3.07 ± 0.59^a^
FSN	1	2	2	2	1	0	1
Aroma	3	3	1	1	2	2	2
Brix (°)	14.17^ab^	13.92^abd^	12.31^c^	13.47^bd^	12.69^cd^	14.47^abd^	13.27^bcd^
*Fom* 0	R	S	R	R	R	S	R
*Fom* 1	R	R	R	R	R	S	R
*Fom* 2	R	R	R	R	R	R	R
MSNV	R	S	S	R	S	S	R
*Px* 1	R	R	R	R	R	S	R
*Px* 2	R	R	R	R	R	S	R
*Px* 3	R	S	R	R	R	S	R
*Px* 3-5	R	R	R	R	R	S	R
*Px* 5	R	R	R	R	R	S	R

*Values followed by a letter presented significantly differences (*P* < 0.05).*

### Pollination With Irradiated Pollen and Parthenogenetic Embryo Rescue

Seven to eight plants of each genotype were grown in the greenhouse to be used as donor plant material (Figure 1A). A total of 1,128 flowers were pollinated with irradiated pollen and 178 of them developed melon fruit (Figures 1C,D). After pollination, some flowers initially developed but later failed to fruit set and finally aborted. A previous experiment had been carried out to analyze the germination of the irradiated pollen and the ability to fruit set. The irradiated pollen germinated correctly and set melon fruit when the female flower was pollinated the same and the next day upon irradiation. More days of storage or different storages reduced dramatically the germination of pollen and the fruit set (data not shown). Analyzing the seven genotypes, significant differences (*P* < 0.05) were found for melon fruit set between genotypes using a Chi Square test. The PS-0301 genotype had the highest fruit set (24.6%) and PS-0709 the lowest (9.7%) (Table 2). Melon fruits of 3 weeks old since pollination were collected and opened for parthenogenetic embryo rescue. Three different protocols were assayed to seek parthenogenetic embryos: one-by-one, X-ray radiography and floating seeds in liquid medium (Table 3). The seeds of 28 melon fruits were opened using the one-by-one method and eight parthenogenetic embryos were found. On the other hand, 127 melons were opened by X-ray radiography and 44 parthenogenetic embryos were found. Finally, 23 melons were opened by floating seeds in liquid medium and one parthenogenetic embryo was found. The percentage of detected melons carrying parthenogenetic embryos was similar between one-by-one and X-ray methods, 28.57 and 34.65%, respectively. Nevertheless, X-ray method was found to be four to five-times faster than one-by-one method due to seeds containing parthenogenetic embryo were the only ones opened (Figures 1E,F). When floating seeds in liquid medium, only 4.35% of melon fruits were contained parthenogenetic embryos due to many of the cultured seeds in liquid medium were contaminated and therefore discarded, despite the initial decontamination of seeds with bleach. Overall, a total of 53 parthenogenetic embryos (Figure 1G) were rescued from the 178 melon fruits set (Table 2). Parthenogenetic embryos were rescued in all donor genotypes, the highest number of parthenogenetic embryos found per genotype was 16, in Melito and the lowest 3, in PS-1901. The ratio of parthenogenetic embryos rescued per melon fruit ranged between 0.14 and 0.38. Although all melon fruits carried a normal number of seeds (between 300 and 500) the 71.91% of fruits had no parthenogenetic embryos. The 53 haploid embryos were recovered from 50 melon fruits. No significant differences (*P* > 0.05) were found using a Chi Square test for the number of parthenogenetic embryos among genotypes.

**TABLE 2:** Parthenogenetic efficiencies of the seven genotypes of melon “Piel de Sapo.”

Genotype	Pollinated flowers	Parthenogenesis induction with irradiated pollen				Germination and *in vitro* growth		Ploidy-level					
		Percentage of developed flowers*	Melon fruits	Embryos	Embryos/melon fruit	Percentage of mortality	Embryos survived	*n*	(%)	2*n*	(%)	*n*/2*n*	(%)
PS-1305	165	10.30	17	6	0.35	33.33	4	3	75	1	25	0	0
PS-1901	143	15.38	22	3	0.14	33.33	2	1	50	1	50	0	0
PS-0301	130	24.62	32	12	0.38	75	3	2	66.67	1	33.33	0	0
PS-0709	196	9.69	19	5	0.26	40	3	3	100	0	0	0	0
PS-2001	169	18.34	31	6	0.19	33.33	4	2	50	2	50	0	0
Melito	202	17.82	36	16	0.44	56.25	7	5	71.43	1	14.29	1	14.29
PS-2301	123	17.07	21	5	0.24	40	3	3	100	0	0	0	0
Total	1,128	15.78*	178*	53	0.30*	50.94	26	19	73.08	6	23.08	1	3.84

Parameters followed by * are significantly different (*P* < 0.05) between genotypes.

**TABLE 3:** Parthenogenetic embryo rescue methods assayed for embryo detection and rescue.

Method	Melons	Embryos	Ratio of fruits
detection	opened	rescued*	containing embryo
One-by-one	28	8	0.28
X-Ray	127	44	0.34
Liquid medium	23	1	0.04

Parameters followed by * are significantly different (*P* < 0.05) between methods.

The parthenogenetic embryos rescued were transferred to solid E20A medium for germination and further plantlet development (Figures 1H,I). From 53 embryos, six failed to germinate, appearing a necrosis in the cotyledonary embryos at the second or third week since rescue. From the 47 germinated embryos, 21 plantlets suffered a stagnation of development and died (Table 2). No significant differences were found between genotypes for embryo germination and plantlet development (*P* > 0.05). Thus, 26 parthenogenetic independent-genotypes plantlets were able to grow *in vitro* and micropropagation was carried out until greenhouse acclimation (Figure 1J).

### Ploidy-Level and Chromosome Doubling

In order to maximize the number of DH plantlets and to ensure the recovery of seeds from DH genotypes, ploidy-level of the parthenogenetic germinated plantlets was analyzed prior to chromosome doubling. Parthenogenesis was found to be successful since haploid, spontaneous DH and mixoploid plantlets were recovered (Table 2). The ploidy-level of the 26 parthenogenetic lines was analyzed by flow cytometry and showed that the 73% were haploid (Figure 1M), the 23% were spontaneous DH (Figure 1N), and one plantlet was found to be mixoploid (Figure 1O). The six spontaneous DH plants were acclimatized in the greenhouse, if they produced pollen and no chromosome doubling was applied. The mixoploid line presented a high ratio of haploid male flowers without pollen and was treated as a haploid.

Chromosome doubling of the 20 haploid parthenogenetic plants was done using different protocols to establish the most efficient one (Table 4). Two antimitotic compounds, colchicine and oryzalin, were assayed for *in vitro* chromosome doubling. On the other hand, colchicine was used for *in vivo* chromosome doubling. A total of 114 nodules or shoot tips were treated *in vitro*, 67 and 47, for colchicine and oryzalin, respectively. *In vitro* colchicine treatment resulted in a high number of dead nodules and shoot tips, 86.57%. From the survived plantlets, only two were successfully chromosome doubled and survived the acclimatazion. *In vitro* oryzalin treatment had a lower short-term death, 89.36% of the nodules or shoot tips survived the next 2 weeks since the chromosome doubling treatment and developed two to three new leaves. Nevertheless, from the 42 survived nodules and shoot tips, 41 presented a high level of hyperhidricity in the base of the nodules or shoot tips that impeded root growing and therefore, no acclimatazion was possible. Final rate of mortality was 95.74%. Only one plantlet was successfully chromosome doubled and acclimatized.

**TABLE 4:** Chromosome doubling protocols assayed.

Treatment			Explants or apical meristems treated	Mortality (%)*	Ploidy-level						Acclimated plants	Melon fruits recovered
Antimitotic compound	Concentration (mg L^–1^)	Time (h)			*n*	%	*n*/2*n*	%	2*n*	%		
Colchicine *in vitro*	500	12	67	86.57	7	36.84	12	63.16	0	0	2	0
Oryzalin *in vitro*	50	18	47	95.74	1	50	0	0	1	50	1	0
Colchicine *in vivo*	5,000	2	150	42.67	45	70.31	6	9.38	13	20.31	–	12

Parameters followed by * are significantly different (*P* < 0.05) between methods.

Due to the low values of the chromosome doubling *in vitro* treatments, *in vivo* chromosome doubling was assayed. Haploid plantlets were acclimatized in the greenhouse (Figure 1K). *In vivo* chromosome doubling was done using colchicine as the antimitotic agent. A total of 150 plant tips were treated with colchicine and 57.33% survived the treatment. From survived plants, 69.77% remained haploid, 9.30% were mixoploids and 20.93% successfully chromosome doubled. The ploidy of chromosome doubled plants, which was analyzed by flow cytometry, was re-checked phenotypically to uphold the successful duplication of plants by checking the presence of pollen in male flowers.

### DH Seed Recovery and Pollen Counts

To recover DH seed, spontaneous DH lines and chromosome doubled lines were autopollinated (Figure 1L). The 33% of DH lines presented male flowers with pollen together with haploid male flowers without pollen, those plants were classified as phenotypically mixoploid although being detected as pure DH by flow cytometry. No fruit recovery was possible from the *in vitro* chromosome doubled plants. From the *in vivo* duplicated plants, a total of twelve melon fruits were recovered from eight independent parthenogenetic DH lines (Table 5). A total of 372 female flowers were pollinated and the fruit set was 3.23%. Genotypes from which no melon fruit was recovered fruit set was impossible. Three out of twelve melon fruits, DH2-PS-2001 and DH8-Melito, carried empty seeds. One chromosome doubled plant, DH4-Melito, did not develop male neither female flowers and no pollination was possible. Finally, DH seed was obtained from six DH plants: DH11-PS-1305, DH3-PS1901, DH9-DH0301, DH5-PS0709, DH10-Melito, and DH1-PS-2301.

**TABLE 5:** Melon fruits recovered from DH lines.

Doubled haploid line	Number of clones	Pollinated flowers	Phenotypical ploidy	Pollen observations	Melon fruits
DH11-PS-1305	5	58	Diploid	Normal male flowers	1
DH3-PS1901	3	47	Diploid	Normal male flowers	2
DH9-DH0301	4	62	Diploid	Small size male flowers with less pollen	1
DH5-PS0709	2	28	Diploid	Normal male flowers	1
DH2-PS-2001	2	36	Diploid	Indehiscent pollen	2
DH8-Melito	3	42	Diploid	Normal male flowers	1
DH10-Melito	3	54	Mixoploid	Normal and haploid male flowers	1
DH1-PS-2301	4	45	Diploid	Normal male flowers	3

## Discussion

Doubled haploid technology has entailed a great progress in plant breeding because of the production of homozygous lines in a shorter time compared to traditional breeding. In *Cucurbitaceae*, DHs are usually produced for commercial means, either to be used as homozygous stable cultivars or as parental pure lines for hybrid F1 seed production. Thanks to heterosis, hybrid F1 cultivars have enhanced traits than their own parental lines. In this work, the donor material was a batch of seven genotypes of melon “Piel de Sapo” type evaluated and characterized for their agronomic traits and pathogen resistances. Later on, their parthenogenetic potential was evaluated focusing on pollination with irradiated pollen, parthenogenetic embryo rescue, *in vitro* plantlet performance and chromosome doubling. The production and the consumption of melon “Piel de Sapo” type is localized mainly in Spain due to cultural reasons where it has a high commercial value because of its differentiated quality. Besides, Spain is the eighth country in terms of melon fruit production worldwide and is the first country in terms of exportation to Europe. We attempted to obtain DH lines of melon with the aim to use them as parental donor lines for commercial hybrid F1 cultivars.

The agronomic traits of melon fruits and the pathogen resistances of the donor material were evaluated in order to analyze the potential use of parthenogenetic-derived DH lines as parental for melon “Piel de Sapo” hybrid F1 cultivars. The agronomic results showed a great variability of melon fruit parameters between the seven evaluated genotypes. Although Melito inbred cultivar presented low pathogen resistances its melon fruits were valuable because of its small dimensions and the high aroma and sucrose content. Monoecious plants are more likely to have elongated fruits (Robinson, 2000), and PS-0301 genotype was monoecious and presented more elongated fruits in comparison to the other six genotypes. The °Brix and aroma of melon fruits were not always correlated. Flavor depends upon taste (sweetness and acidity) and aroma. Besides, °Brix only measures the concentration of predominant sugars, as fructose, sucrose and glucose, and organic acids. Aroma is often considered to play a dominant role in flavor of fruits and vegetables and is dependent upon low-molecular-weight-volatile compounds as largely esters, alcohols, aldehydes and ketones, which are not measured with the refractometer (Kader, 2008). Therefore, melon fruits of PS-1305, PS-1901, and Melito were considered as the best ones in terms of balance between aroma and sugar content. The majority of genotypes assayed presented pathogen resistances. Pathogen resistance or susceptibility to *Podosphaera xhantii*, causing powdery mildew, *Fusarium oxysporum* f.sp. *melonis*, causing fusarium wilt, and MNSV was evaluated because are the major diseases in melon. The use of resistant cultivars is the best approach to control pathogen spreading and disease. In the southern of Europe, *Podosphaera xhantii* races 1, 2, and 5 are the most frequent (Yuste-Lisbona et al., 2010). Although powdery mildew can be controlled by fungicides its long-term use led to fungicide resistance of powdery mildew. The use of resistant cultivars is a more effective and environmentally safe way to control the disease. On the other hand, *Fusarium oxysporum* f.sp. *melonis* is one of the most difficult diseases to control because the pathogen is soil-borne and remains viable in the soil as chlamydospores (Joobeur et al., 2004). Concerning to MNSV, the best source of resistance in melon is the *nsv* gene, which confers a recessive resistance to MNSV (Nieto et al., 2007). Therefore, genotypes such as PS-1305, PS-0709, and PS-2301, were considered as the best genotypes it terms of pathogen resistance.

The genotype of the donor material has a crucial influence for the success of DH protocols as reported in many species, including melon. Parthenogenesis in *inodorus* “Piel de Sapo” type genotypes has been reported once and had not been much studied in comparison to other genotypes such as *inodorus* “Galia” type, *chinensis* or *cantalupensis*, possibly because of its local importance. In this parthenogenetic study, the genotypic response of seven genotypes of melon “Piel de Sapo” type differed for: fruit set when pollinated with irradiated pollen; parthenogenetic embryo induction; haploid embryo germination; chromosome doubling; and, fruit set of DH lines. The parthenogenetic ability of melon “Piel de Sapo” germplasm used was lower than other genotypes such as *chinensis*, *cantalupensis* or *inodorus* (Lotfi et al., 2003; Lim and Earle, 2008; Gonzalo et al., 2011).

Fruit set of donor plants after pollination with irradiated pollen is the first step of *in situ* parthenogenesis. In *Cucurbitaceae*, *in situ* parthenogenesis induction through gamma-ray irradiated-pollen has been achieved in melon, cucumber, watermelon and winter squash since the first report of Sauton and Dumas de Vaulx (1987). Nevertheless, no reports focus on the efficiency of the pollination with irradiated pollen. In this study, the number of pollinated flowers with irradiated pollen and its later development or drop was recorded. The efficiency of pollination varied between genotypes, the lowest value was 9.69% and the highest 24.62%, in PS-0709 and PS-0301, respectively. The low number of developed female flowers (15.78% in average) and fruit set is attributable to the irradiation process suffered by the pollen. Although irradiated pollen can germinate on the stigma and grow within the style reaching the embryo sac is genetically inactivated to fertilize the egg-cell and the polar nuclei. Therefore, irradiated pollen stimulates egg-cell division and induces haploid embryos (Cuny, 1992). Pollen sensitivity to irradiation its attributed to radio-resistance, and the viability of pollen is decreased along with the irradiation exposure. Previous reports in melon (Lim and Earle, 2008; Gonzalo et al., 2011; Godbole and Murthy, 2012) used an irradiation exposure of 250 Gy, therefore, prior to the experiment, this dose was evaluated based on fruit set and pollen germination assays (data not shown). Moreover, during pollination with irradiated pollen, fruit set was observed to be dependent on: the time of the year, being August the period when more fruit set; the stage of donor plants, at the beginning of flowering and the end of the greenhouse culture fruit set was low; and, the weather, cloudy pollination days resulted in less fruits than shinny days. Pollen storage viability was evaluated through the pollination with irradiated male flowers with zero, one and two days since irradiation. The storage of irradiated male flowers in plastic pots in darkness for one day was successful to maintain pollen viability. Therefore, pollen could be used to pollinate female flowers. More than one day of storage resulted in a decrease of pollen viability and inability to set melon fruits.

The parthenogenetic embryo production was reported to be genotypic dependent. From all genotypes, a total of 178 melon fruits and 53 embryos were obtained. Normally, the parthenogenetic embryo efficiency is expressed as embryos per seed. Nevertheless, the process of detecting embryos is tedious enough to additionally count the seeds. In this study, the efficiency was expressed as parthenogenetic embryos contained per fruit. The ratio of embryos per fruit in melon “Piel de Sapo” ranged between 0.14 and 0.44, similar to the 0–3 reported in genotypes of “Piel de Sapo” by Gonzalo et al. (2011). Besides, it was lower than the ratio of 4–18 in *inodorus* genotype of the “Galia” type reported by Lotfi et al. (2003) and the high ratio of 16 reported by Lim and Earle (2008). About one third of melon fruits contained at least one embryo, meaning that the vast majority of melon fruits had an average of 400 empty seeds. The parthenogenetic embryo detection process is laborious and time-consuming, the results are very inefficient compared with the time invested. Because of this, different methodologies have been described to detect parthenogenetic embryos, being the inspection of seeds one-by-one the most commonly applied (Chun et al., 2006; Smiech et al., 2008; Godbole and Murthy, 2012), followed by X-ray radiography of seeds (Dolcet-Sanjuan et al., 2004; Claveria et al., 2005) and the culture of seeds in liquid medium (Lotfi et al., 2003). In this study, three methods were assayed in order to reduce the amount of time and work invested during the process of embryo detection without compromising the embryo itself. Although seed culture in liquid medium reduced drastically the amount of work it was not effective and compromised the parthenogenetic embryo because of endophytic bacterial and fungi contaminations, despite the initial sterilization of seeds. On the other hand, one-by-one and X-ray methods resulted in a similar ratio of embryo per melon, 0.28 and 0.35, showing that both did not compromise the obtention of embryos. Nevertheless, X-ray method was five time faster than one-by-one method. Then, X-ray method was selected for routine laboratory use.

Once parthenogenetic embryos were detected, they were cultured *in vitro* for germination, development and micropagation. From 53 rescued embryos, 26 germinated, grew *in vitro* and developed plantlets. The 11.32% of embryos failed to germinate and from those germinated, the 39.62% died before the first micropropagation was possible because failed to grow and did not develop the first true leaf. *In vitro* germination and growth are critical steps that can jeopardize the *in situ* parthenogenetic process. Deleterious gene combination in homozygosity regulating vegetative growth may be responsible of hampering germination and plantlet development (Geoffriau et al., 1997). During the *in vitro* process there is a high selection pressure impeding the survival of embryos with deleterious recessive alleles in homozygosity (Cuny, 1992). The results showed a mortality of 25–66.67% depending on the genotype and is in accordance with other authors, reporting a 42–62% (Lim and Earle, 2008) or 62–84% (Lotfi et al., 2003).

To restore diploid chromosome content in haploid melon plants, induced chromosome doubling is mandatory. In cucurbits, the number of spontaneous doubled haploids obtained during DH methodology is usually low compared with other species, that can represent the 30–55% of androgenetic plants in bell pepper (Irikova et al., 2011; Keleş et al., 2015) or 30% in rice (Hooghvorst et al., 2018). The ploidy-level of the produced plants was analyzed: 73% were haploid, 4% mixoploid and 23% spontaneous chromosome doubled. The ploidy-level results presented are in accordance with those of Lim and Earle (2008), who found a 73% of haploids and 27% mixoploids in melon; or Kurtar and Balkaya (2010), that produced 76.71% spontaneous DHs and 23.29% haploids in squash; or, Sauton (1988, 1989), who reported spontaneous doubling in cucumber and melon for the first time. Spontaneous duplication can occur when endomitosis or nuclear fusion happens. In endomitosis process, cell multiplicate chromosomes and separate them in each cell pole during early mitosis, nevertheless, cell fails to divide, and two sets of chromosomes is restituted. In nuclear fusion, two or more synchronized nuclei divide and develop a common spindle (Kasha, 2005). Spontaneous DHs and mixoploids have an endomitotic or nuclei fusion origin. Notwithstanding, in spontaneous DHs, the endomitosis or nuclear fusion took place at early stages of the development of the egg-cell, and in mixoploid plants occurred later, causing a different ploidy-level of the germ cells. Then, induced chromosome doubling of haploid plants is necessary prior to DH seed recovery. Colchicine is the most used antimitotic for chromosome doubling in DH technology. When haploids are treated with antimitotic compounds a so-called C-mitosis can take place. During interphase, cells have their chromosomes duplicated with the chromatid sisters placed in each pole of the cell bound by the centromere’ spindle tubule. The antimitotic compound interacts with the tubulin subunits and destabilize the spindle tubule arresting cells during interphase. Chromosome doubling is a required step in parthenogenesis DH protocols in *Cucurbitaceae*. Three different protocols were assayed for chromosome doubling, two protocols *in vitro* with colchicine or oryzalin, and one *in vivo* with colchicine. *In vitro* protocols resulted in a high mortality rate due to antimitotic toxicity. The recorded chromosome duplication efficiency of 500 mg L^–1^ of colchicine for 12 h *in vitro* treatment was 63.16%. However, majority of plantlets failed to develop after the treatment and before the ploidy-level analysis. The rate of chromosome doubled and successfully acclimatized plants was 2.98% (two plants). Similar results were recorded for 50 mg L^–1^ of oryzalin for 18 h *in vitro* treatment which resulted in one plant (2.13%) survived and chromosome doubled. Those results show a high sensitivity of the genotypes to the antimitotic compounds and to the *in vitro* culture once treated, which do not line with other reported studies that show *in vitro* chromosome doubling treatment as successful and the preferred (Dong et al., 2016; Ebrahimzadeh et al., 2018). This could be explained as a recalcitrant performance of the “Piel de Sapo” genotypes not only to the antimitotic treatment but also to *in vitro* culture, which in turn resulted in a low production of DH lines. Due to the low efficiency of survival and chromosome doubling of *in vitro* treatments, *in vivo* 5,000 mg L^–1^ of colchicine for 2 h on apical meristems was assayed. Resulting in 69.77% of survival and 13.33% of chromosome doubling, being 8% DHs and 5.33% mixoploids. Other authors have reported higher chromosome duplication efficiencies when treating *in vivo* with colchicine as 46.03% in “Kirkagac” and “Yuva Hasanbey” melon genotypes (Solmaz et al., 2011) or 19% (Lim and Earle, 2008). The *in vivo* chromosome doubling efficiencies were acceptable in spite of being low, those are in line with the low *in vitro* efficiencies and other parameters analyzed previously, which support the hypothesis of the recalcitrant performance of “Piel de Sapo” genotypes during the entire parthenogenetic process. The fruit set of the 20 DH lines and the eight mixoploid lines was low. A total of 12 fruits from eight independent parthenogenetic DHs were recovered. Pollination of induced chromosome doubled plants was dramatically difficult, an average of 3.23% of pollinated female flowers set fruit. This ratio was even lower than the pollination with irradiated pollen. From the fruits recovered, both of DH2-PS-2001 and the one of DH10, contained the usual amount of seeds despite all of them were empty. Consequently, DH seed was not recovered from those genotypes. Lim and Earle (2008) proved that pollen viability of chromosome doubled plants its usually low, affecting fruit set and seed viability. In their study, they recommend *in vitro* chromosome doubling because had higher pollen germination rates than *in vivo* chromosome doubled plants. We had no success in *in vitro* chromosome doubling, and the *in vivo* chromosome doubled plants had a low capacity to set fruits.

In this study, “Piel de Sapo” donor material had traits with potential value for commercial purposes such as melon fruit morphology, sweetness and aroma, and pathogen resistances against important diseases such as powdery mildew, fusarium wilt and MNSV virus. Moreover, the *in situ* parthenogenetic capacity of “Piel de Sapo” germplasm was evaluated, showing: a low capacity of fruit set when pollinated with irradiated pollen, a low production of parthenogenetic embryos, a poor *in vitro* culture performance, a low chromosome doubling and a low fruit set of DH lines once chromosomes were doubled. The “Piel de Sapo” *inodorus* type can be considered as a recalcitrant genotype for parthenogenesis in melon species in comparison to other genotypes. Nevertheless, we succeed to obtain DH seed that have a great value for hybrid F1 seed production and commercialization. During the parthenogenetic process, X-ray method was concluded as the most successful and optimum method to detect and rescue parthenogenetic embryos. The poor performance of “Piel de Sapo” genotypes showed during *in vitro* culture could be enhanced changing media composition instead of using the traditional E20A medium. In addition, an *in vivo* chromosome doubling method with colchicine was adapted and resulted as the most successful for chromosome doubling of haploid plants, in front of *in vitro* chromosome doubling methods with colchicine or oryzalin. Although parthenogenetic DH plants were obtained from six out of seven melon “Piel de Sapo” genotypes further improvements of the process using variations should be assayed in order to produce a higher number of DH plants that could be used in melon breeding programs.

## Data Availability Statement

All datasets generated for this study are included in the article/supplementary material.

## Author Contributions

IH designed, supervised, and participated in all the experiments and wrote the manuscript. OT participated during the first stage of parthenogenesis process and helped writing the manuscript. SH participated and supervised the agronomic and pathogen evaluation experiments and corrected the manuscript. SN helped during all parthenogenetic process and manuscript correction.

## Conflict of Interest

The authors declare that the research was conducted in the absence of any commercial or financial relationships that could be construed as a potential conflict of interest.
